# Influence of Tamoxifen on Different Biological Pathways in Tumorigenesis and Transformation in Adipose-Derived Stem Cells, Mammary Cells and Mammary Carcinoma Cell Lines—An In Vitro Study

**DOI:** 10.3390/cells11172733

**Published:** 2022-09-01

**Authors:** Frederik Schlottmann, Vesna Bucan, Sarah Strauß, Felix Koop, Peter M. Vogt, Tobias R. Mett

**Affiliations:** Department of Plastic, Aesthetic, Hand and Reconstructive Surgery, Hannover Medical School, Carl-Neuberg-Strasse 1, 30625 Hannover, Germany

**Keywords:** mamma carcinoma, breast cancer, tamoxifen, adipose derived stem cells, ASC, autologous fat grafting, breast reconstruction, PCR array, tumorigenesis

## Abstract

Breast carcinoma is one of the most common malignant tumors in women. In cases of hormone-sensitive cells, tamoxifen as an anti-estrogenic substance is a first line medication in the adjuvant setting. The spectrum of autologous breast reconstructions ranges from fat infiltrations to complex microsurgical procedures. The influence of adipose-derived stem cells (ASC) on the tumor bed and a possibly increased recurrence rate as a result are critically discussed. In addition, there is currently no conclusive recommendation regarding tamoxifen-treated patients and autologous fat infiltrations. The aim of the present study was to investigate the effect of tamoxifen on the gene expression of a variety of genes involved in tumorigenesis, cell growth and transformation. Mammary epithelial cell line and mammary carcinoma cell lines were treated with tamoxifen in vitro as well as co-cultured with ASC. Gene expression was quantified by PCR arrays and showed increased expression in the mammary carcinoma cell lines with increasing time of treatment and concentration of tamoxifen. The data presented can be considered as an addition to the controversial discussion on the relationship between ASC and breast carcinoma cells. Further studies are needed to quantify the in vivo interaction of ASC and mammary carcinoma cells and to conclusively assess the impact of tamoxifen in reconstructive cases with fat grafting.

## 1. Introduction

With over two million new cases and nearly 630,000 deaths in 2018, breast cancer is the most occurring cancer in women around the world [1]. Due to the high prevalence, nationwide mammography screening is recommended. Following the diagnosis, early therapeutic approaches recommended by guidelines combine breast-conserving operations, radiotherapy and anti-estrogen therapy for estrogen receptor (ER) positive breast cancer for up to five years following the operation [2]. Curative surgical approaches range from breast-conserving procedures to gland ablation with combined sentinel lymph node biopsy [3]. Complete tumor resection with tumor-free surgical margins is highly relevant to prognosis and long-term survival [4]. Due to the increased survival after curative therapy of breast carcinoma, there is an increasing patient request for reconstructive plastic surgery procedures for breast reconstruction [5]. Although breast-conserving operations have rapidly improved, a large percentage of treated patients are unsatisfied with the aesthetic outcomes [3,6]. In particular, a mastectomy is considered to be devastating physically and psychologically [7]. The broad spectrum of reconstructive options ranges from alloplastic materials, such as tissue expanders and mammary implants, to autologous tissue transfer with numerous flap plasty techniques [7]. Furthermore, autologous fat grafting has become globally accepted and is routinely performed for aesthetic and reconstructive reasons [8].

Due to the diversity of reconstructive options and the patient’s individual risk profile, a joint decision regarding the surgical procedure should be made after a detailed consultation [9]. In addition to surgical treatment options, neo-adjuvant or adjuvant radiation is of great importance for locoregional tumor control and the prevention of local recurrence [10]. Following breast-conserving operations, radiotherapy is mandatory according to current guidelines [11]. The need and the type of adjuvant breast cancer drug therapy mainly depends on tumor size, lymph node status, grading, hormone-receptor status, human epidermal growth factor 2 (HER2) status, menopausal status and age [12,13]. Furthermore, the amplification rate of Ki-67 plays a crucial role in the classification of tumor subtypes and the decision to adopt a possible adjutant drug system therapy [12]. Systemic chemotherapy can be administrated in a neo-adjuvant and adjuvant setting and should be based on anthracycline and taxane [14]. In addition, for HER2-positive breast carcinomas, systematic therapy with trastuzumab should be considered [15]. Patients with ER and progesterone receptor (PR)-positive invasive mamma carcinomas should be considered for systemic treatment with tamoxifen besides any potential chemotherapy [16]. However, tamoxifen therapy should be started once the chemotherapy has finished, but can be carried on parallel to radiotherapy [17].

By the competitive inhibition of the ER, tamoxifen modulates genes, for example, genes encoding growth factors and pro-angiogenic factors, that are regulated by estrogen [16]. Tamoxifen is referred to as a selective ER modulator because of its anti-estrogenic and estrogenic properties, but the full molecular biological basis for these properties is not completely understood yet [18]. Tamoxifen is meant to slow down cell proliferation in breast cancer tissue and can cause tumor regress [16]. An influence of direct programmed cell death induced by tamoxifen has also been described in literature [19]. As a result, adjuvant tamoxifen treatment reduces the risk of local and contralateral recurrence in breast intraepithelial neoplasia [20]. Long-term data also demonstrated that tamoxifen use over 5 years in patients with ER-positive mamma carcinoma reduces the absolute risk of recurrence from 4.8% to 2.9% [21,22]. Besides the modulating effects on ER, recent studies have shown a cytotoxic effect of tamoxifen on ER-negative cells [23]. These are thought to be initiated by a reduced glutamine uptake and elevated oxidative stress, resulting in apoptosis in in vitro trials [23]. However, the systematic effect of tamoxifen on ER negative cells is not fully understood to date.

As mentioned above, autologous fat grafting plays a crucial role in the contour restoration of the breast, in addition to aligning mastopexies and reduction mammoplasties of the opposite side [24]. Autologous fat grafting is also known to improve tissue damage caused by radiotherapy, treat chronic pain after breast-conserving surgery and improve wound healing in general [25,26]. However, the relationship between autologous fat grafting and breast cancer recurrence remains controversial. On the one hand, a recent study reported no increased risk of tumor recurrence or new tumor development after a 10 year follow-up of patients treated with autologous fat grafting for breast reconstruction [27]. On the other hand, another study reported a slightly higher risk of local tumor recurrence, although not statistically significant, after autologous fat grafting for breast reconstruction compared to the untreated control group [28].

In autologous fat grafting, adipose-derived stem cells (ASC) contained in the adipose tissue are also transferred from the donor side to the oncological operated breast [29]. ASC are multipotent adipose-derived stem cells with the potential to form bone, cartilage, muscle and fat tissue, representing an exciting perspective for regenerative medicine and surgery [30,31,32]. It was described earlier that ASC contribute to fat tissue turnover and enhance vascularization [33,34]. Furthermore, electron microscopy showed the presence of ASC around the endothelial cells of small vessels in human breast adipose tissue [35]. It was shown earlier that transplanted ASC can maintain the viability of fat transplants through the secretion of growth factors and improve tissue survival [36]. However, it remains unclear whether grafted or resident ASC may increase the risk of de novo carcinoma development in the breast or local recurrence [29]. Many studies focused on the relationship of adipocytes and breast cancer cells, showing that mature adipocytes metabolize androgen to estrogen through aromatase activity, thereby affecting mamma carcinoma cell growth by paracrine mechanisms [37]. As mammary adipose tissue is one of the main sites for estrogen biosynthesis, its local production and delivery is likely to be involved in cancer progression [38]. It was earlier shown that murine and human mature adipocytes co-cultured with mammary carcinoma cells exhibited changes in the number and size of lipid droplets, decreased adipose tissue specific markers and over-expressed interleukin-6 levels, leading to a more aggressive tumor behavior [39]. However, the detailed molecular biological interactions and relationships between mature adipocytes and breast cancer cells are not conclusively understood yet and are still critically discussed [29].

So far, only a few studies have investigated the influence of tamoxifen therapy on ASC, and the results remain controversial. On the one hand, Mandlekar et al. showed that tamoxifen induces caspase-dependent apoptosis [40]. Other studies confirmed these results, showing that tamoxifen inhibits ASC proliferation and induces apoptosis in a dose and time-dependent manner [41]. On the other hand, Boemi et al. pointed out that the concentration of tamoxifen is much lower in fat than in other parts of the body and claimed that tamoxifen does not significantly affect cellular functions and apoptosis rates [42]. However, the effect of tamoxifen on the gene expression of ASC involved in fat graft survival, tumorigenesis and the transition of mammary carcinoma cells has not been analyzed yet.

The present study aimed to investigate the effect of tamoxifen on the gene expression of a wide variety of genes involved in cell growth, tumorigenesis and transformation. Information about the proliferative characteristics of fat grafts regarding the survival and stimulation of ACS should help health care specialists involved in adjuvant and reconstructive therapy after breast cancer.

## 2. Materials and Methods

### 2.1. Isolation and Cell Culture of ASC

Human ASC were isolated from adipose tissue after abdominoplasty surgery from a female donor aged 35 years, with informed written and oral consent of the patient and approval by the Ethics Committee of Hannover Medical School (protocol code: 3475-2017; date of approval: 15 February 2017). The isolation and characterization of ASC were performed according to standardized methods described earlier [31]. All experiments were performed according to internal standardized protocols and followed good manufacturing practice. ASC were cultured in Dulbecco’s modified Eagle’s growth medium (DMEM-F12) without calcium/magnesium (PAA Laboratories, Pasching, Austria), adding 10% (*v*/*v*) fetal bovine serum (FBS) (Sigma-Aldrich, Merck, Darmstadt, Germany) and 50 mg/mL ascorbic acid-2-phosphate (Sigma-Aldrich, Merck, Darmstadt, Germany). ASC were incubated at 37 °C and 5% CO_2_ in a humidified atmosphere, and cell culture medium was changed thrice weekly. Upon reaching confluence, cells were detached with 0.05% (*v*/*v*) trypsin/ethylenediamine tetraacetic acid (T/EDTA) (Gibco, ThermoFisher Scientific, Waltham, MA, USA) and subcultured. ASC of passage 2 were used for all of the following experiments.

### 2.2. Cell Culture of MCF-10A Mammary Cell Line

Human MCF-10A cell line, as a non-tumorigenic mammary epithelial cell line, was commercially available (American Type Culture Collection, Manassas, VA, USA). According to the manufacturers’ instructions, cells were derived from a female donor aged 36 years, and the cell line was produced by long-term culture in serum-free medium with low calcium concentration. According to the manufacturers’ instructions, MCF-10A cells were cultured in DMEM-F12 containing 5% (*v*/*v*) FBS (Sigma-Aldrich, Merck, Darmstadt, Germany), 100 ng/mL Cholera toxin (Sigma-Aldrich, Merck, Darmstadt, Germany) and 10 μg/mL insulin (Biochrome, ThermoFisher Scientific, Waltham, MA, USA). MCF-10A cells were incubated at 37 °C and 5% CO_2_ in a humidified atmosphere, and the cell culture medium was changed thrice weekly. Upon reaching confluence, cells were detached with 0.05% (*v*/*v*) T/EDTA (Gibco, ThermoFisher Scientific, Waltham, MA, USA) and subcultured. MCF-10A cells of passage 29 were used for all of the following experiments.

### 2.3. Cell Culture of MCF-7 Mammary Carcinoma Cell Line

Human MCF-7 cell line, as a mammary adenocarcinoma cell line, with ER positivity, was commercially available (LGC Standards, Wesel, Germany). According to the manufacturers’ instructions, cells were derived from a female Caucasian donor aged 69 years and retained several characteristics of differentiated mammary epithelium including the ability to process estradiol via cytoplasmic estrogen receptors and the capability of forming domes. MCF-7 cells were cultured in RPMI-1640 medium (Gibco, ThermoFisher Scientific, Waltham, MA, USA) supplemented with 10% (*v*/*v*) FBS (Sigma-Aldrich, Merck, Darmstadt, Germany), 25 mM HEPES (Biochrome, ThermoFisher Scientific, Waltham, MA, USA), 1 mM sodium pyruvate (Biochrome, ThermoFisher Scientific, Waltham, MA, USA) and 10 μg/mL insulin (Biochrome, ThermoFisher Scientific, Waltham, MA, USA). MCF-7 cells were incubated at 37 °C and 5% CO_2_ in a humidified atmosphere, and cell culture medium was changed thrice weekly. Upon reaching confluence, cells were detached with 0.05% (*v*/*v*) T/EDTA (Gibco, ThermoFisher Scientific, Waltham, MA, USA) and subcultured. MCF-7 cells of passage 32 were used for all of the following experiments.

### 2.4. Cell Culture of BT-474 Mammary Carcinoma Cell Line

Human BT-474 cell line, as a mammary epithelial carcinoma cell line, with HER2 receptor positivity, was commercially available (CLS Cell Line Service, Eppelheim, Germany). According to the manufacturers’ instructions, cells were derived from a female donor aged 60 years. In 1978, E. Lasfargues and W.G. Coutinho isolated the BT-474 cell line from a solid, invasive ductal carcinoma of the breast [43]. BT-474 cells were cultured in DMEM-F12 supplemented with 5% (*v*/*v*) FBS (Sigma-Aldrich, Merck, Darmstadt, Germany) and 5 µg/mL insulin (Biochrome, ThermoFisher Scientific, Waltham, MA, USA). BT-474 cells were incubated at 37 °C and 5% CO_2_ in a humidified atmosphere, and cell culture medium was changed thrice weekly. Upon reaching confluence, cells were detached with 0.05% (*v*/*v*) T/EDTA (Gibco, ThermoFisher Scientific, Waltham, MA, USA) and subcultured. BT-474 cells of passage 15 were used for all of the following experiments.

### 2.5. Co-Culture of Cells

To generate the co-cultures, 1 × 10^6^ cells per cell population were selected and co-cultured in a 1:1 ratio. Thus, three co-cultures were generated: ASC and MCF-10A, ASC and MCF-7, and ASC and BT-474. The cell culture medium used was composed of 50% ASC medium (see Section 2.1.) and 50% cell type-specific medium, as mentioned in Section 2.2, Section 2.3, and Section 2.4. Cells were incubated at 37 °C and 5% CO_2_ in a humidified atmosphere, and cell culture medium was changed thrice weekly. Upon reaching 75–85% confluence, cells were treated with tamoxifen for 48 or 96 h.

### 2.6. Tamoxifen Treatment of Cells

Tamoxifen citrate, as potent synthetic anti-estrogen, was commercially available (Calbiochem, Merck, Darmstadt, Germany; # 579000). According to the manufacturers’ instructions, 50 mg of tamoxifen citrate was dissolved in 10 mL pure ethanol (J.T. Baker, ThermoFisher Scientific, Waltham, MA, USA). Tamoxifen solution was added to the cell culture medium to treat the cells. The four cell populations (ASC, MCF-10A, MCF-7 and BT-474) were each treated individually, as well as the three co-cultures. Cells were treated with a final concentration of 5 µM, 15 µM and 25 µM tamoxifen for 48 and 96 h and cell culture medium containing tamoxifen was changed daily. Untreated samples served as control.

### 2.7. RNA Extraction and Quantitative Real-Time-PCR Array

After 48 or 96 h of treatment with tamoxifen, cells were washed twice with phosphate-buffered saline (PBS) (Gibco, ThermoFisher Scientific, Waltham, MA, USA). The total RNA was extracted using TRIzol™ reagent (Invitrogen, ThermoFisher Scientific, Waltham, MA, USA) according to the manufacturers’ instructions. RNA concentration was quantified using a NanoDrop™ One photospectrophotometer (ThermoFisher Scientific, Waltham, MA, USA) and samples were normalized to 1 µg total RNA. Reverse transcription was performed with 1 µg total RNA using the iScriptTMcDNA Kit (Bio-Rad Laboratories, Hercules, CA, USA) according to the manufacturers’ instructions. To analyze the influence of tamoxifen on nine different biological pathways involved in transformation and tumorigenesis, the Human Cancer PathwayFinder RT^2^ Profiler PCR Array (PCR array) (Qiagen, Venlo, Netherlands; Cat. #330231, GeneGlobe ID PAHS-033ZA) was used according to the manufacturers’ instructions. Using the PCR array, real-time polymerase chain reaction (PCR) can easily and reliably analyze the expression of a focused panel of 84 genes related to oncogenesis. Table 1 gives an overview of the examined 84 genes as well as their biological pathway functions. The PCR array was performed following the manufacturers’ protocol. The three steps of the cycling program were 95 °C for 10 min, then 95 °C for 15 s, 55 °C for 40 s and 72 °C for 30 s. The steps were repeated for 40 cycles using the Bio-Rad iCycler (Bio-Rad Laboratories, Hercules, CA, USA). All reactions were performed using SsoFast EvaGreen Supermix (Bio-Rad Laboratories, Hercules, CA, USA) in a total volume of 15 μL. The relative expression intensity was determined by calculating the 2-Δ-cycle threshold (Ct) for each sample. All samples were normalized to the used housekeeping genes actin, beta-2-microglobulin, and glyceraldehyde 3-phosphate dehydrogenase. The Ct values of the untreated samples were set to zero. Relative gene expression was calculated from the established zero line using Microsoft Excel software version 2016 (Microsoft Cooperation, Redmond, WA, USA). The resulting higher or lower expressions of the studied genes were represented by different color nuances. A relatively lower Ct value indicated earlier expression of the studied gene compared to controls. In contrast, a higher Ct value indicated decreased expression.

## 3. Results

### 3.1. PCR Array of MCF-10A Showed Increased Gene Expression after Tamoxifen Treatment and Co-Cultures with ASC Showed Decreased Gene Expression

**Figure 1:**
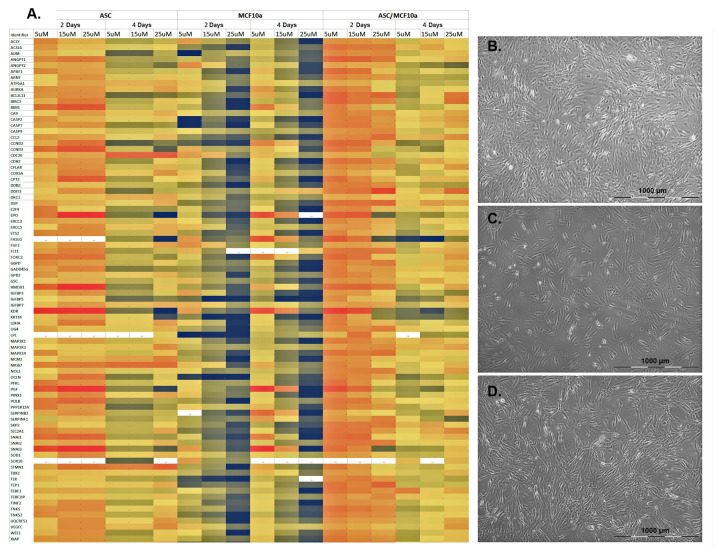
Gene expression of the examined genes of the PCR arrays of ASC, MCF-10A cells and corresponding co-cultures. (**A**) From left to right, ASC, MCF-10A cells and co-culture were each treated with 5 µg, 15 µg or 25 µM tamoxifen for 48 or 96 h. The color-coding (see Figure 2) indicates a relative increase (black) or reduction (red) in gene expression. The 84 genes studied and their associated pathways in tumorigenesis and transformation are listed in Table 1. (**B**) ASC of passage 2 after treatment with 25 µM tamoxifen after 96 h in culture. (**C**) MCF-10A cells of passage 29 after treatment with 25 µM tamoxifen after 96 h in culture. (**D**) Co-culture of ASC and MCF-10A cells after treatment with 25 µM tamoxifen after 96 h in culture.

Human ASC were readily isolated, classified and subcultured (Figure 1B). There were also no abnormalities with regard to the cell culture of MCF-10A (Figure 1C). After reaching a confluence of 75–85%, the cells were stimulated individually with tamoxifen at a concentration of 5 µM, 15 µM and 25 µM for a period of 48 or 96 h with daily treatment with tamoxifen. In addition to treating the individual cells with tamoxifen, the MCF-10A cells were co-cultured with ASC (Figure 1D) and also treated with tamoxifen according to the above given regimen.

**Figure 2:**
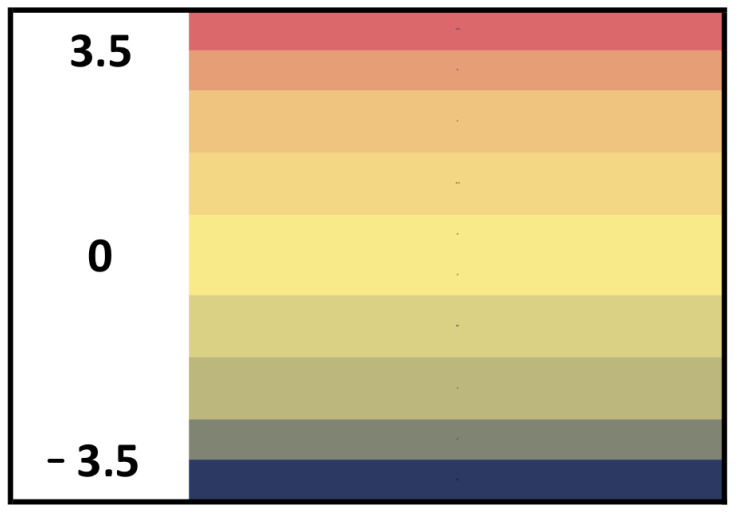
Legend of the color-coding of Figure 1, Figure 3 and Figure 4. Negative values (black color-coding) indicate a relative reduction of the Ct value and thus increased gene expression. Positive values (red color-coding) indicate a relative increase in Ct values and thus reduced gene expression.

After 48 and 96 h, PCR arrays for gene expression analysis were performed. Untreated cells served as controls in each experimental group (data not shown). The relative gene expression intensity was determined by calculating each sample’s 2-Δ-Ct. All samples were normalized to the used housekeeping genes actin, beta-2-microglobulin and glyceraldehyde 3-phosphate dehydrogenase. The Ct values of the untreated samples were set to zero, and relative gene expression was calculated from the established zero line. The resulting higher or lower expressions of the studied genes were represented by different color nuances, as shown in Figure 2.

A relatively lower Ct value indicated earlier expression of the studied genes compared to controls. In contrast, a higher Ct value indicated decreased expression. Therefore, negative values (black color-coding) indicate a relative reduction of the Ct value and thus increased gene expression. Positive values (red color-coding) indicate a relative increase in Ct values and thus reduced gene expression. Figure 1A shows the results of the 84 genes examined in the PCR array of ASC and MCF-10A cells.

Treatment of a pure culture of ASC with 5 µM tamoxifen showed the reduced expression of all genes examined after a period of 48 h (Figure 1A, left column). With an increase of the tamoxifen concentration up to 25 µM, the overall trend of gene expression continued to decrease after 48 h, as is visible from the increasing red color-coding (Figure 1A, left column). Interestingly, an increase in the duration of treatment with tamoxifen to 96 h showed an increased expression of the genes, recognizable by the black color-coding. However, an increase of the tamoxifen concentration to 25 µM rather showed a relative decrease of gene expression again (Figure 1A, left column). Treatment of a pure culture of MCF-10A cells with 5 µM tamoxifen for 48 h showed a slight overall increase in gene expression of the genes studied. This effect could be further increased by increasing the tamoxifen concentration to 25 µM (Figure 1A, middle column). In contrast, prolonging the tamoxifen treatment to 96 h showed no difference in the results after 48 h, even with increasing concentrations (Figure 1A, middle column). Treatment of co-cultures of ASC and MCF-10A cells showed overall reduced gene expression of genes following treatment with 5 µM tamoxifen over a 48 h period. Here, an increase in tamoxifen concentration to a 48 h period showed nearly constant gene expression (Figure 1A, right column). In contrast, the treatment of the co-cultures with tamoxifen over 96 h showed a rather decreasing gene expression, with relative values around the defined zero line (Figure 1A, right column). In summary, the treatment of mammary epithelial MCF-10A cells with tamoxifen increased the expression of the 84 genes studied, whereas co-cultures with ASC tended to show reduced gene expression overall.

### 3.2. Co-Cultures of MCF-7 Mammary Carcinoma Cell Line and ASC Showed Increased Gene Expression with Increasing Tamoxifen Concentrations after 48 h

**Figure 3:**
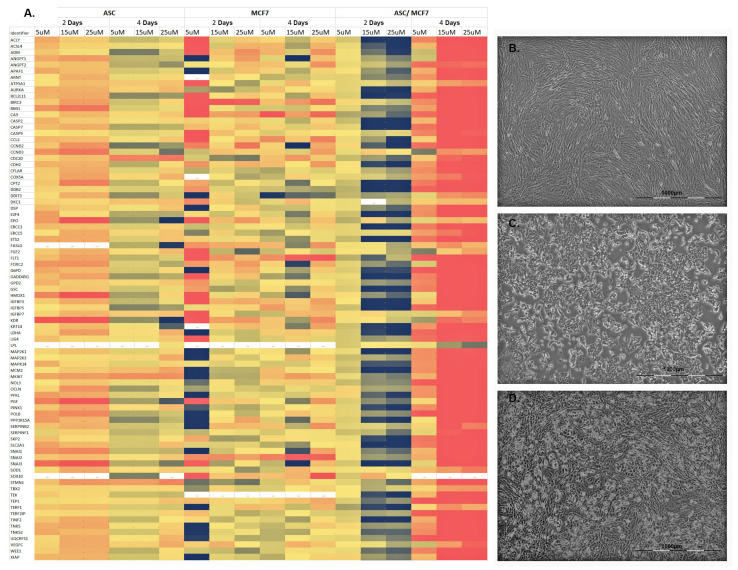
Gene expression of the examined genes of the PCR arrays of ASC, MCF-7 cells and corresponding co-cultures. (**A**) From left to right, ASC, MCF-7 cells and co-culture were each treated with 5 µg, 15 µg or 25 µM tamoxifen for 48 or 96 h. The color coding (see Figure 2) indicates a relative increase (black) or reduction (red) in gene expression. The 84 genes studied and their associated pathways in tumorigenesis and transformation are listed in Table 1. (**B**) ASC of passage 2 after treatment with 25 µM tamoxifen after 96 h in culture. (**C**) MCF-7 cells of passage 32 after treatment with 25 µM tamoxifen after 96 h in culture. (**D**) Co-culture of ASC and MCF-7 cells after treatment with 25 µM tamoxifen after 96 h in culture.

With regard to the ASC pure cultures treated with tamoxifen, the results obtained after 48 and 96 h could be reproduced, as is recognizable from the identical color coding of Figure 1A, left column, and Figure 3A, left column. Interestingly, the treatment of MCF-7 cells with 5 µM tamoxifen after 48 h showed both increased and decreased expression of genes of all pathways, meaning that no clear trend of expression could be detected here (Figure 3A, middle column). However, with an increase of the tamoxifen concentration up to 25 µM over a period of 48 h or prolonging the treatment to 96 h, the gene expression of all pathways around the zero line could be detected again (Figure 3A, middle column).

By co-culturing ASC and MCF-7 cells, an increased expression of the genes of all pathways was observed after 48 h at a concentration of tamoxifen of 15 µM as well as 25 µM (Figure 3A, right column). However, prolonging the treatment with tamoxifen to 96 h showed a significant reduction of the investigated genes at all tamoxifen concentrations. In summary, an increase in the gene expression of all pathways involved in tumorigenesis and transformation was detected in co-cultures of ASC and MCF-7 cells after treatment with tamoxifen over 48 h.

### 3.3. Co-Culture of BT-474 Mammary Carcinoma Cell Line and ASC Showed a Moderate Increase in Gene Expression of Pathways Involved in Tumorigenesis and Transformation

**Figure 4:**
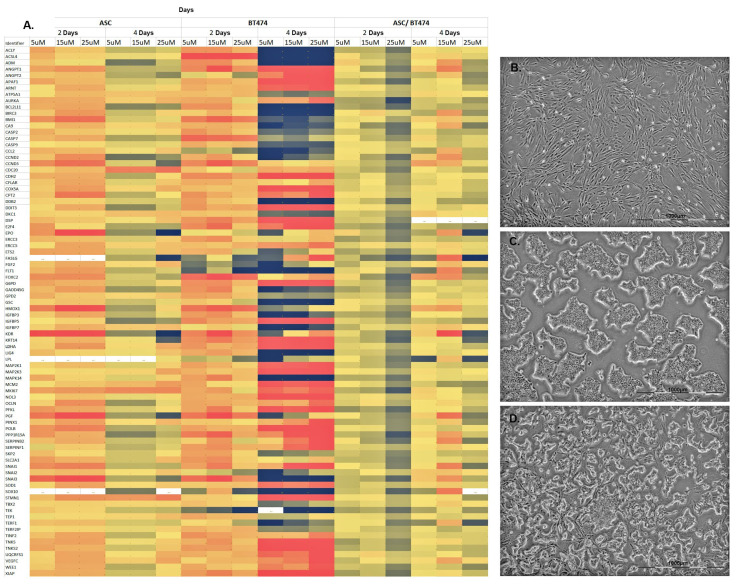
Gene expression of the examined genes of the PCR arrays of ASC, BT-474 cells and corresponding co-cultures. (**A**) From left to right, ASC, BT-474 cells and co-culture were each treated with 5 µg, 15 µg or 25 µM tamoxifen for 48 or 96 h. The color coding (see Figure 2) indicates a relative increase (black) or reduction (red) in gene expression. The 84 genes studied and their associated pathways in tumorigenesis and transformation are listed in Table 1. (**B**) ASC of passage 2 after treatment with 25 µM tamoxifen after 96 h in culture. (**C**) BT-474 cells of passage 15 after treatment with 25 µM tamoxifen after 96 h in culture. (**D**) Co-culture of ASC and BT-474 cells after treatment with 25 µM tamoxifen after 96 h in culture.

Concerning the ASC pure cultures treated with tamoxifen, the results obtained after 48 and 96 h could be reproduced, as is recognizable from the identical color coding of Figure 1A, left column, and Figure 4A, left column. Treatment of BT-474 cells with 5 µM tamoxifen for 48 h resulted in an overall reduced expression of all involved genes. Only a few genes showed a relative increase in gene expression (Figure 4A, middle column). Increasing the tamoxifen concentration did not result in any significant changes in gene expression over 48 h. By extending the treatment of BT-474 cells with tamoxifen to a period of 96 h, a colorful expression pattern of the involved genes of all pathways was revealed. Overall, both increased and decreased expressions of a wide variety of genes of the different pathways were observed, meaning that no clear trend could be identified (Figure 4A, middle column). An increase in tamoxifen concentration showed constant expression patterns after 96 h.

By co-culturing ASC and BT-474 cells, an increased expression of the genes of all pathways was observed after 48 h at a concentration of tamoxifen of 15 µM as well as 25 µM (Figure 4A, right column). Prolonging the treatment with tamoxifen to 96 h showed a reduction of the investigated genes at all tamoxifen concentrations compared to the values at 48 h. In summary, an increase in gene expression of all pathways involved in tumorigenesis and transformation was detected in a co-culture of ASC and BT-474 cells after treatment with tamoxifen over 48 h with decreasing expressions after 96 h.

### 3.4. Comparison of 14 Genes with the Strongest Expression Variation

**Figure 5:**
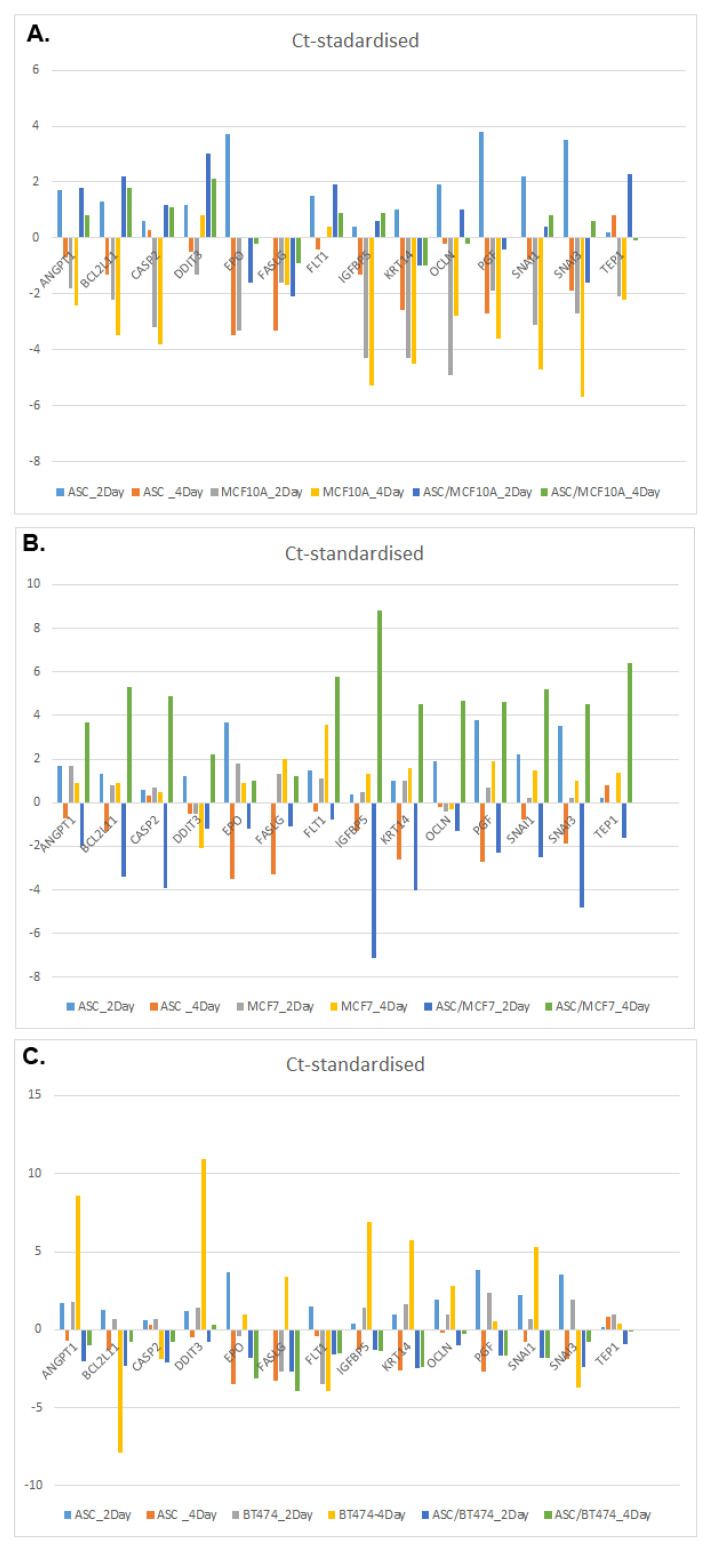
Expression patterns of the 14 genes with the strongest variations. For an overview of the gene abbreviations, please refer to Table 1. A positive value indicates a higher Ct value and thus down-regulated gene expression. A negative value means earlier and thus increased gene expression. (**A**) Expression patterns of ASC, MCF-10A cells and co-cultures. (**B**) Expression patterns of ASC, MCF-7 cells and co-cultures. (**C**) Expression patterns of ASC, BT-474 cells and co-cultures.

To further quantify the results of the genes examined from the PCR arrays, 14 genes with the greatest variation in expression and their associated pathways were identified and presented separately. Table 2 provides a corresponding overview. Figure 5 shows the deviations of the relative expression from the standardized Ct values in the form of bar charts. As in the previous experiments, a positive value indicates a higher Ct value and thus down-regulated gene expression. A negative value means earlier and thus increased gene expression.

Figure 5A shows the gene expression of the genes listed in Table 2 in ASC, MCF-10A cells as well as in the corresponding co-cultures, each after a period of 48 and 96 h after tamoxifen treatment with 25 µM. A pure culture of ASC showed significantly reduced expression of EPO after 48 h of tamoxifen treatment. However, this effect was reversed after 96 h of tamoxifen treatment with a significantly increased EPO expression in ASC. A pure culture of MCF-10A cells and co-cultures of ASC and MCF-10A cells showed increased EPO expression after 48 and 96 h. ANGPT1 showed a similar expression pattern with reduced gene expression after 48 h of tamoxifen treatment in a pure culture of ASC (Figure 5A). However, increasing the treatment duration to 96 h showed increased ANGPT1 expression, as well as in MCF-10A cells treated for 48 or 96 h. However, co-cultures of ASC and MCF-10A cells again showed decreased expression of ANGPT1 at 48 and 96 h. In contrast, FASLG showed increased gene expression in all cultures at all time points (Figure 5A). IGFBP5 exhibited reduced gene expression in ASC after 48 h of tamoxifen treatment but showed significantly increased gene expression after 96 h of tamoxifen treatment and in pure cultures of MCF-10A cells. However, co-culturing of ASC and MCF-10A cells resulted in reduced expression of IGFBP5 after tamoxifen treatment (Figure 5A). PGF and SNAI3 exhibited similar expression behavior with initially significantly reduced gene expression after 48 h of tamoxifen treatment in a pure culture of ASC. After 96 h of tamoxifen treatment, the pure cultures of ASC and MCF-10A cells after 48 and 96 h showed a significantly increased gene expression of PGF and SNAI3. Co-culturing of ASC and MCF10A cells then again showed reduced gene expression of PGF and SNAI3 (Figure 5A).

Treatment of ER-positive MCF-7 cells and ASC with 25 µM tamoxifen showed other genes to be the most affected regarding the greatest variation in expression. For example, BCL2L11 in a pure culture of ASC initially showed decreased gene expression after 48 h of tamoxifen treatment but a marked increase in expression with increase in treatment time to 96 h (Figure 5B). MCF-7 cells showed decreased expression of BCL2L11 after both 48 and 96 h of tamoxifen treatment. Co-cultures of ASC and MCF-7 cells showed a markedly increased expression of BCL2L11 after 48 h of tamoxifen treatment, which reversed to the opposite after prolonging the treatment duration to 96 h. The pure cultures of ASC again showed reduced gene expression of EPO after 48 h of tamoxifen treatment, with an increase in expression after 96 h. In the co-cultures of ASC and MCF-7 cells, the expression of EPO initially increased again after 48 h but decreased after prolonging the duration of treatment with tamoxifen to 96 h. Overall, all 14 genes initially showed increased gene expression in the co-cultures of ASC and MCF-7 cells after 48 h of treatment with tamoxifen. However, this regressed for all genes, prolonging the treatment time to 96 h, and showed an opposite effect with decreased gene expression. BCL2L11, CASP2, IGFBP5 and SNAI3 showed the greatest variations in expression (Figure 5B).

Figure 5C shows the gene expression of the genes listed in Table 2 in ASC and HER2-positive BT-474 cells, as well as in the corresponding co-cultures, each after a period of 48 and 96 h after tamoxifen treatment with 25 µM. Regarding ANGPT1 expression, the pure cultures of ASC showed comparable expression results to the preliminary experiments in Figure 5A,B. However, BT-474 cells showed decreased expression of ANGPT1 by tamoxifen treatment after 48 h, which further decreased after 96 h (Figure 5C). The co-cultures of ASC and BT-474 cells showed significantly increased expression of ANGPT1. The expression of BLC2L11 showed comparable results in ASC pure cultures after 48 and 96 h of tamoxifen treatment compared to Figure 5A,B. Treatment of BT-474 cells with tamoxifen for 48 h initially showed decreased gene expression of BLC2L11. However, after prolonging the tamoxifen treatment to 96 h, significantly increased gene expression is shown. Co-cultures of ASC and BT-474 cells also showed increased gene expression of BCL2L11 after 48 and 96 h of tamoxifen treatment (Figure 5C). DDIT3 initially showed decreased and minimally increased expression in ASC after 48 and 96 h of tamoxifen treatment, respectively. In BT-474 cells, DDIT3 expression was considerably decreased and even significantly increased by prolonging the treatment duration with tamoxifen from 48 to 96 h. The co-cultures then showed minimally increased gene expression again after 48 h of treatment, but this regressed after 96 h of tamoxifen treatment (Figure 5C). In addition, IGFBP5 showed comparable results in terms of gene expression in the ASC pure cultures. The BT-474 cells showed significantly decreased expression of IGFBP5 after tamoxifen treatment at 48 and 96 h (Figure 5C). However, by co-culturing ASC and BT-474 cells, an increased expression of IGFBP5 was shown, which even minimally increased by prolonging the treatment duration with tamoxifen (Figure 5C). Overall, it is of interest that all 14 genes showed an increase in expression in co-cultures of ASC and BT-474, recognizable by the negative dark blue and green bar graphs (Figure 5C).

## 4. Discussion

To the authors’ knowledge, the present study is the first of its kind to investigate the changes in gene expression of pathways of cell growth, tumorigenesis and transformation in ASC, MCF-7, MCF-10A and BT-474 cells at the biomolecular level. In hormone therapy for ER-positive mamma carcinoma, tamoxifen as a synthetic non-steroidal anti-estrogen remains the drug of choice in premenopausal patients [16]. In patients at high risk of recurrence, oral tamoxifen medication is recommended to block ER for at least 5 years postoperatively [44]. Permitting once-daily administration, tamoxifen is absorbed readily after oral administration, and serum half-lives and its major metabolites range from 7 to 14 days [45,46]. However, it was reported that concentrations of tamoxifen and its active metabolites vary from patient to patient and tissue to tissue [45]. The highest concentrations were found in lung and liver, being 8 to 70-fold higher than in serum [47]. The concentrations fluctuate during one dosing interval in most tissues, except fat and testes, in which tamoxifen concentrations are relatively stable [47]. Therefore, in the present study, cells were treated with tamoxifen once daily, as in the clinical setting. In addition, a concentration series was established to provide information on the concentration and time-dependent change in the gene expression studied. The interaction of tamoxifen in a complex tissue, as would be expected in an in vivo setting, as well as the interaction, e.g., with other drugs, such as erythromycin, cyclosporine, nifedipine and diltiazem [48], is left out in the experimental design of the present study and requires further in vivo experiments in the future.

Besides the modulating effects on ER, recent studies have shown a cytotoxic effect of tamoxifen on ER-negative cells [23]. Thus, the data to date regarding the influence of tamoxifen on ASC are limited and partly very controversial. In particular, conflicting data regarding the role of ASC in cancer progression were reported, showing that in in vivo and in vitro experiments, ASC favored tumor growth and increased extracellular matrix deposition and vascularization [49]. On the one hand, some studies investigated the effect of tamoxifen in vitro on ASC and demonstrated that tamoxifen concentrations above 2 µM had cytotoxic effects on ASC in terms of apoptosis induction and proliferation inhibition [41]. They also showed that tamoxifen appears to decrease the multipotent potential capacity of ASC [41]. These findings were further outlined by Madelkar et al., who showed that high concentrations of tamoxifen can induce caspase-dependent apoptosis [40], which could not be observed in this study. On the other hand, further studies showed controversial results. In contrast to the present results, it was postulated that tamoxifen does not affect cellular proliferation, vascular endothelial growth factor secretion and apoptosis of ASC [42]. Furthermore, the gene expression assessment demonstrated no impairment in the differentiation capacity of ASC after tamoxifen treatment [42]. The results of the study at hand are another keystone to depict the controversial facts about ASC and parallel tamoxifen treatment. No proliferation arrest or increased apoptosis was detected by treating ASC with different concentrations of tamoxifen for a period of 48 and 96 h. However, the present study saw decreased gene expression of CASP2, CASP7 and CASP9 after tamoxifen treatment for 48 h, with increased gene expression at 96 h. Thus, increased caspase-dependent apoptosis of ASC would be possible in the course beyond 96 h, as described by Pike et al. [41]. In future studies, an extension of the study period is needed to quantify the previous results.

The ASC used in the study at hand were isolated from adipose tissue after abdominoplasty surgery from a female donor aged 35 years. It was reported that patient factors can influence the multipotent capacity and viability of ASC [50]. In particular, increasing age, body mass index, diabetes mellitus and radiotherapy, as well as tamoxifen treatment, are described as potential influencing factors, although the latter was not uniformly seen across all studies [50]. In future studies, the PCR arrays should therefore be extended to a larger patient population as well as supplemented with ASC from tamoxifen-treated patients to further place the present study data in the scientific context of the published literature.

In addition to the effect of tamoxifen on ASC, the interaction between ASC and mammary carcinoma cells has also been extensively studied. Overall, conflicting evidence emerged during recent years about the safety profile of ASC applications as ASC may induce tumor progression and invasion [49]. In this respect, it is still unclear whether preadipocytes act differently from mature adipocytes [33]. Deriving from the secretory activity of ASC with the secretion of chemokines, growth factors and other various cytokines, pro and anti-oncogenic effects of ASC were postulated [49,51]. By secreting more than 474 proteins, ASC influence their local tissue environment as well as a possible tumor environment [51]. It is believed that the compositions and concentrations of soluble factors secreted by the ASC are among the most important mediators of tumor progression and invasion [52]. It was postulated that the secretory activity of ASC affected normal mammary epithelial cells as well as mammary carcinoma cells [53]. Co-cultures of ASC and different human mamma carcinoma cell lines were performed and showed concomitantly up-regulated tumor-associated genes and their corresponding proteins [54]. As published, co-cultures of ASC and MCF-7 cells fostered the proliferation of MCF-7 [55]. The results indicated that grafted ASC favor the growth of active but not dormant tumor cells; moreover, transplantation or co-injection into a mouse breast cancer model did not promote tumor growth or metastasis [56]. Another study showed that ASC in co-culture with epithelial breast cancer cells undergoing epithelial-to-mesenchymal-transition showed mesenchymal features with loss of polarity and a stem cell-like spindle shape that favors motility, invasiveness and survival [57]. In this context, ASC may interact with breast cancer cells through the formation of gap junctions that allow intercellular communication and the exchange of molecular weight compounds [58,59]. As described in other studies, the presence of gap junctions correlates with a more malignant phenotype and greater tumor progression and thereby can modulate the metastatic potential of the breast cancer cells [60]. Another study discovered the link between a cytoskeleton-based pathway in ASC and the stromal impact on breast cancer cells [39]. Overall, the current scientific evidence and data regarding the relationship between ASC and mammary carcinoma cells is inconsistent and at times contradictory. In the present study, pure cultures of ASC, MCF-10A, MCF-7 and BT-474 cells were prepared and co-cultured. There were no abnormalities with regard to the cell culture of all cells. After reaching a confluence of 75–85%, the cells were stimulated individually with tamoxifen at a concentration of 5 µM, 15 µM and 25 µM for a period of 48 or 96 h with daily treatment with tamoxifen. Untreated cells served as controls in each experimental group and defined the zero line to compare altered gene expression by tamoxifen treatment. Overall, the data collected can thus be seen as complementary to the published literature.

MCF-10A is a non-tumorigenic mammary epithelial cell line that has the characteristics of normal breast epithelium indicated by the lack of tumorigenicity in nude mice, three-dimensional growth in collagen, growth in culture that is controlled by hormones and growth factors, lack of anchor-age-independent growth and dome formation in confluent cultures [61]. In the present study, the treatment of MCF-10A cells with tamoxifen increased the expression of the 84 genes studied, whereas co-culture with ASC tended to show reduced gene expression overall. A pure culture of ASC showed significantly reduced expression of EPO after 48 h of tamoxifen treatment. However, this effect was reversed after 96 h of tamoxifen treatment with a significantly increased EPO expression in ASC. A pure culture of MCF-10A cells, as well as co-cultures of ASC and MCF-10A cells, showed increased EPO expression after both 48 and 96 h. EPO, as a marker of the hypoxia signaling pathway, encodes the protein erythropoietin, which is secreted into the blood circulatory system and targets erythroid progenitor cells in the bone marrow to stimulate blood production [62]. However, treating breast cancer patients with EPO has been associated with poor prognosis and decreased survival [63]. Furthermore, EPO induced the epithelial-to-mesenchymal transition in MCF-10A, showing the process in which epithelial mammary cells were transdifferentiated to a mesenchymal state [64]. This development should therefore be considered critical since the pathway is associated with tumor progression, as epithelial cells acquire the ability to carry out the various steps of the invasion or metastasis process through transition. Thus, the emergence of a malignant tumor from MCF-10A cells could be hypothetically possible. The extent to which ASC can potentiate this process cannot yet be conclusively assessed and requires further studies.

Treatment of ASC cells with tamoxifen showed similar expression patterns of ANGPT1 with reduced gene expression after 48 h of tamoxifen. However, increasing the treatment duration to 96 h showed increased ANGPT1 expression, as well as in MCF-10A cells treated for 48 or 96 h. However, a co-culture of ASC and MCF-10A cells again showed decreased expression of ANGPT1 at 48 and 96 h. ANGPT1 encodes for the protein angiopoietin-1, which is known as an angiogenic promoter in embryonic angiogenesis by promoting vascular branching, pericyte recruitment and endothelial survival [65]. It can be stated that prolonged tamoxifen treatment over 96 h or co-culturing ASC and MCF-10A with tamoxifen treatment leads to decreased expression of pro-angiogenic factors. To what extent this influences the reduced vascularization of fat grafts in clinical application should be investigated in further in vivo studies. However, the data situation of the literature remains controversial as another study showed that tamoxifen reduced the rates of resorption and fibrosis of the injected fat, resulting in better integration of the autologous fat graft [66]. It is of interest that in the present study, FASLG showed increased gene expression in all cultures at all time points. FASLG encodes for a transmembrane protein that induces T cell-mediated apoptosis [67]. Thus, increased apoptosis of both ASC and MCF-10A and their respective co-cultures would be expected but could not be observed within the present study. To what extent this impacts clinical applications in the context of autologous fat grafting remains to be investigated in further in vivo studies. Again, clinical experience showed no correlation between tamoxifen treatment and decreased take rates of autologous fat grafts [66].

MCF-7 is known as an epithelial adenocarcinoma mammary cell line that expresses ER [68,69]. Interestingly in the study at hand, treatment of MCF-7 cells with 5 µM tamoxifen after 48 h showed both increased and decreased expression of genes of all pathways, so no clear trend of expression could be detected. However, with an increase of the tamoxifen concentration up to 25 µM over a period of 48 h or after prolonging the treatment to 96 h, the gene expression of all pathways around the zero line could be detected again. An increase in the gene expression of all pathways involved in tumorigenesis and transformation was detected in a co-culture of ASC and MCF-7 cells after treatment with tamoxifen over a period of 48 h. Co-culture of ASC and MCF-7 cells showed markedly increased expression of BCL2L11 after 48 h of tamoxifen treatment, which reversed to the opposite with an increase in treatment duration to 96 h. Similar effects could be demonstrated for EPO, IGFBP5 and SNAI 3. BCL2L11 is known as an apoptotic activator through interactions with various members of the BCL-2 protein family [70].

Thus, based on the gene expression pattern, it can be stated that co-culturing ASC and MCF-7 cells initially induces increased apoptosis at 48 h. However, after 96 h of tamoxifen treatment, the gene expression of this pro-apoptotic protein is reduced, meaning that an increased proliferation of MCF-7 cells would potentially be conceivable. However, this has to be seen in the context of the complexities of the mediators involved in apoptosis to make a final decision [71]. The expression results of EPO, IGFBP5 and SNAI3 are similar.

Regarding the results of the expression of EPO, the presented results are contradictory to the published literature. It was described elsewhere that recombinant human erythropoietin altered gene expression and stimulated the proliferation of MCF-7 cells [72]; however, the MCF-7 cells were not treated with tamoxifen. IGFBP5 encodes for a protein of apoptotic cells that increases the adhesion of epithelial cells on a mesenchymal but not epithelial extracellular matrix [73]. It was shown earlier that IGFBP5 induced cell adhesion, increased cell survival and inhibited cell migration in MCF-7 cells. Based on the results of this study, decreased gene expression of IGFBP5 should thus induce decreased cell adhesion and increased apoptosis as well as the cell migration of MCF-7 cells by co-culturing with ASC and tamoxifen treatment. Hypothetically, this could lead to increased tumor growth clinically. Again, the clinical results contradict the molecular biological data of the present study [66].

BT-474 cells are known as an immortalized epithelial mammary carcinoma cell line that expresses HER2 receptor positivity [43]. HER2 expression has been associated with poor prognosis and decreased patient survival [74,75]. There is substantial evidence that HER2 positivity affects various anti-cancer treatments such as hormone therapy with tamoxifen, radiotherapy and cytokine therapy [75]. HER2 overexpression is also associated with anti-estrogen resistance and thereby reduces effectivity of tamoxifen treatment [76]. Thus, BT-474 cells should not be accessible to tamoxifen unless combined with trastuzumab, as described elsewhere [77]. It is of interest that in the present study, almost all genes studied showed an increase of expression in co-cultures of ASC and BT-474 after treatment with tamoxifen. For example, significantly increased expression of BCL2L11 would lead to increased apoptosis and thus decreased tumor growth. DDIT3 encodes a protein that has pro-apoptotic transcription factor properties [78]. Thus, the reduced expression would lead to reduced induced apoptosis and thus potentially to increased tumor growth.

In contrast, increased FASLG expression would also be expected to increase cell apoptosis. Increased EPO expression, on the other hand, would suggest increased tumor vigilance, which would be consistent with data in the previous literature [79]. It remains to be seen to what extent the in vitro data obtained can reflect a changed clinical outcome. However, comprehensive further in vitro and in vivo studies are needed to further quantify the in vitro data with regard to gene expression.

The available data spark controversy, as clinical studies do not report elevated tumor incidence or recurrence rates after the application of ASC. However, tamoxifen medication might be a crucial factor for cell growth in general and cancer recurrence. The data in the present study could show that gene expression patterns in co-cultures of ASC and mammary cells for different pathways of tumorigenesis and transformation partly provided controversial results to the previous clinical data. In particular, the significant impact of tamoxifen on BT-474 cells despite the absence of ER expression should not be disregarded here. The broad acceptance of tamoxifen in the adjuvant setting could not be argued on the base of these in vitro results, but a molecular understanding is still warranted. Although a higher take rate of fat graft might be assumed due to the upregulation of proliferative pathways, a discontinuation of tamoxifen medication prior to possible autologous fat grafting should also be kept in mind due to the same effect on cancer cells. A stimulation of ASC to transform in a malignant manner cannot be excluded.

Consequently, the existing data and the present pilot study urge further research to elucidate the previous in vitro findings and place them in the broad context for future clinical applications. A surgeon should critically evaluate the careless administration of ASC on a case-by-case basis, as there are no conclusive recommendations for clinical practice to date.

## Figures and Tables

**Table 1:** Overview of the genes examined and their associated pathways using the Human Cancer PathwayFinder RT^2^ Profiler PCR Array (Qiagen, Venlo, Netherlands). Overall, 84 genes representative of 9 different biological pathways involved in tumorigenesis and transformation were examined. Biological pathways included angiogenesis, apoptosis, cell cycle, cellular senescence, DNA damage and repair, epithelial-to-mesenchymal transition, hypoxia signaling, metabolism and telomere maintenance.

Function	Gene
Angiogenesis	Angiopoetin 1 (ANGPT1) Angiopoetin 2 (ANGPT2) Chemokine (C-C motif) ligand 2 (CCL2) Basic fibroblastic growth factor 2 (FGF2) Kinase insert domain receptor (KDR) Vascular endothelial growth factor receptor 1 (VEGFR1) Vascular endothelial growth factor 3 (VEGFR3) Placental growth factor (PGF) Serpin peptidase inhibitor, clade F (alpha-2 antiplasmin, pigment epithelium derived factor), member 1 (SERPINF1) TEK tyrosine kinase, endothelial (TEK) Vascular endothelial growth factor C (VEGFC)
Apoptosis	Apoptotic peptidase activating factor 1 (APAF1) B-cell lymphoma 2 like 11 (apoptosis facilitator) (BCL2L11) Baculoviral inhibitor of apoptosis repeat-containing protein 3 (BIRC3) Caspase 2, apoptosis-related cysteine peptidase (CASP2) Caspase 7, apoptosis-related cysteine peptidase (CASP7) Caspase 9, apoptosis-related cysteine peptidase (CASP9) Caspase 8 und fas-associating death domain-containing protein-like apoptosis regulator (CFLAR) Fas- ligand (TNF superfamily, member 6) (FASLG) Nucleolar protein 3 (apoptosis repressor with CARD domain) (NOL3) X-linked inhibitor of apoptosis (XIAP)
Cell cycle	Aurora kinase A (AURKA) Cyclin D2 (CCND2) Cyclin D3 (CCND3) Cell division cycle 20 homolog (S. cerevisiae) (CDC20) E2F transcription factor 4, p107/p130-binding (E2F4) FMS related tyrosine kinase 1 (FLT1) Minichromosome maintenance complex component 2 (MCM2) Antigen identified by monoclonal antibody Ki-67 (MKI67) S-phase kinase-associated protein 2 (p45) (SKP2) Stathmin 1 (STMN1) WEE homolog (S.pombe) (WEE1)
Cellular Senescence	BMI1 polycomb ring finger oncogene (BMI1) V-Ets erythroblastosis virus E26 oncogene homolog 2 (avian) (ETS2) Insulin-like growth factor binding protein 3 (IGFBP3) Insulin-like growth factor binding protein 5 (IGFBP5) Insulin-like growth factor binding protein 7 (IGFBP7) Mitogen-activated protein kinase 1 (MAP2K1) Mitogen-activated protein kinase 3 (MAP2K3) Mitogen-activated protein kinase 14 (MAPK14) Serpin peptidase inhibitor, clade B (ovalbumin), member 2 (SERPINB2) Superoxidase dismutase 1, soluble (SOD1) T-box 2 (TBX2)
DNA Damage and Repair	Damage-specific DNA binding protein 2, 48kDa (DDB2) DNA-damage-inducible transcript 3 (DDIT3) Excision repair cross-complementing rodent repair deficiency, complementation group 3 (ERCC3) Excision repair cross-complementing rodent repair deficiency, complementation group 5 (ERCC5) Growth arrest and DNA-damage-inducible, gamma (GADD45G) Ligase IV, DNA, ATP-dependent (LIG4) Polymerase (DNA directed), beta (POLB) Protein phosphatase 1, regulatory (inhibitor) subunit 15A (PPP1R15A)
Epithelial-to-Mesenchymal Transition	Cadherin 2, type 1, N-cadherin (neuronal) (CDH2) Desmoplakin (DSP) Forkhead box C2 (MFH-1, mesenchyme forkhead 1) (FOXC2) Goosecoid homeobox (GSC) Keratin 14 (KRT14) Occludin (OCLN) Snail homolog 1 (Drosophila) (SNAI1) Snail homolog 2 (Drosophila) (SNAI2) Snail homolog 3 (Drosophila) (SNAI3) SRY (sex determining region Y)-box 10 (SOX10)
Hypoxia Signaling	Adrenomedullin (ADM) Aryl hydrocarbon receptor nuclear translocator (ARNT) Carbonic anhydrase IX (CA9) Erythropoietin (EPO) Heme oxygenase (decycling) 1 (HMOX1) Lactate dehydrogenase A (LDHA) Solute carrier family 2 (facilitated glucose transporter), member 1 (SLC2A1)
Metabolism	ATP citrate lyase (ACLY)Acyl-CoA synthetase long-chain family member 4 (ACSL4) ATP synthase, H+ transporting, mitochondrial F1 complex, alpha subunit 1, cardiac muscle (ATP5A1) Cytochrome c oxidase subunit Va (COX5A) Carnitine palmitoyltransferase 2 (CPT2) Glucose-6-phosphate dehydrogenase (G6PD) Glycerol-3-phosphate dehydrogenase 2 (mitochondrial) (GPD2) Lipoprotein lipase (LPL) Phosphofructokinase, liver (PFKL) Ubiquinol-cytochrome c reductase, Rieske iron-sulfur polypeptide 1 (UQCRFS1)
Telomeres and Telomerase	Dyskeratosis congenita 1, dyskerin (DKC1) PIN2/TERF1 interacting, telomerase inhibitor 1 (PINX1) Telomerase-associated protein 1 (TEP1) Telomeric repeat binding factor (NIMA-interacting 1) (TERF1) Telomeric repeat binding factor 2, interacting protein (TERF2IP) TERF1 (TRF1)-interacting nuclear factor 2 (TINF2) Tankyrase, TRF1-interacting ankyrin-related ADP-ribose polymerase (TNKS) Tankyrase, TRF1-interacting ankyrin-related ADP-ribose polymerase 2 (TNKS2)

**Table 2:** Overview of the genes and their corresponding pathways that showed the strongest expression variation.

Function	Gene
Angiogenesis	*ANGPT1*
Apoptosis	*BCL2L11* *CASP2* *FASLG*
Cell Cycle	*FLT1*
Cellular Senescence	*IGFBP5*
DNA Damage and Repair	*DDIT3*
Epithelial-to-Mesenchymal Transition	*KRT14* *OCLN* *SNAI1* *SNAI3*
Hypoxia Signaling	*EPO*
Metabolism	−/−
Telomeres and Telomerase	*TEP1*

## Data Availability

Not applicable.
